# Influence of 8‐week endurance training on resting energy expenditure and body composition in women

**DOI:** 10.14814/phy2.70591

**Published:** 2025-09-30

**Authors:** Ida E. Löfberg, Aino Kuljukka, Vera M. Salmi, Johanna K. Ihalainen, Heikki Kyröläinen, Anthony C. Hackney, Ritva S. Mikkonen

**Affiliations:** ^1^ Faculty of Sport and Health Sciences University of Jyväskylä Jyväskylä Finland; ^2^ Research Institute for Olympic Sport (KIHU) Jyväskylä Finland; ^3^ Department of Exercise & Sport Science–Department of Nutrition University of North Carolina Chapel Hill North Carolina USA; ^4^ Sports Technology Unit, Faculty of Sport and Health Sciences University of Jyväskylä Vuokatti Finland

**Keywords:** body composition, endurance training, menstrual cycle, metabolic hormones, oral contraceptives, resting metabolism

## Abstract

Endurance training potentially induces changes in resting energy expenditure (REE), body composition, and metabolic hormones, but well‐controlled studies accounting for hormonal status of women are sparse on this topic. This study investigated how 8‐week moderate intensity endurance training (MIET) affects these outcomes in recreationally active women. Naturally menstruating women (NoOC, *n* = 17) and combined oral contraceptive users (COC, *n* = 8) were assessed in the luteal/follicular phases and active/inactive phases before and after MIET. REE and respiratory exchange ratio (RER; by indirect calorimetry), body composition, estradiol, progesterone, free triiodothyronine (fT3), acylated and unacylated ghrelin were measured at rest. Linear mixed models with time, group, and their interaction were used to analyze the results in two stratified phase comparisons. No interaction effects were observed, but a main effect of time for RER was observed in the pooled sample, indicating a 5.6% increase (*p* = 0.004) at follicular/inactive phase. The COC group also had higher fT3 concentrations at all time points (*p* < 0.05). Two participants from different groups showed consistent ≥10% REE changes. An 8‐week MIET did not induce detectable changes in REE, body composition, or metabolic hormones on a group level, but high individual variation was observed.

## 1 INTRODUCTION

The female body experiences a coordinated monthly ebb and flow of hormones, which is a unique physiological characteristic that might influence hormonal and metabolic adaptations to exercise. Major strides have been made toward a better understanding of these adaptations in women, but overall research focus in sport and exercise sciences still tends to lean toward male‐centric models (Cowley et al., 2021). Endurance training is defined as repetitive exercise aimed at improving oxygen delivery and aerobic performance (Jones & Carter, 2000). To meet the cumulative demands of increased muscle contractions, multiple physiological systems must adapt to maintain homeostasis (Hawley et al., 2014). Resting energy expenditure (REE), which covers the energy expended for vital body functions (Heymsfield et al., 2021), is particularly sensitive to shifts in energy homeostasis (Siedler et al., 2023). Since REE constitutes ~70% of total daily energy expenditure (Ravussin & Bogardus, 1992), it is a critical component of energy balance. It is also considered a key non‐invasive marker of metabolic adaptation, as it is suggested to respond to both short‐ and long‐term changes in energy balance (Siedler et al., 2023).

Theoretical models based on population‐level studies propose that REE may either increase, decrease, or remain unchanged after increased physical activity energy expenditure (Careau et al., 2021). In practice, it is unclear how REE adapts to endurance training after accounting for changes in fat‐free mass (FFM) and fat mass (FM) (Heymsfield et al., 2021). Most studies investigating the influence of 6 weeks–1 year of moderate‐intensity endurance training (MIET) report inconsistent findings: while most show no marked change in REE (Gomersall et al., 2016; Lee et al., 2009; Scharhag‐Rosenberger et al., 2010; Willis et al., 2014; Wilmore et al., 1998), there are also studies demonstrating decreases or increases in REE (Byrne & Wilmore, 2001; Potteiger et al., 2008). However, previous studies investigating REE in the context of endurance training have typically involved heterogeneous, mixed‐sex populations, with no rigorous control for female‐specific confounding variables such as menstrual cycle and hormonal contraceptive use.

The absence of controlling for the menstrual cycle in studies examining metabolism is a limitation, as the totality of evidence suggests that REE is slightly higher during the luteal phase, when estradiol (E2) and progesterone (P4) are elevated, compared to the early follicular phase, when hormone levels are low (Benton et al., 2020). Leptin, an adipokine involved in energy homeostasis, is suggested to align with the rhythm of E2 and P4, with elevated concentrations in the luteal phase (Salem, 2021). Leptin is known to be involved in the maintenance of the thyroid axis (Flier et al., 2000), whereas thyroid hormones are critical regulators of cellular energy expenditure (Kim, 2008) and are suggested to crosstalk with female sex hormones at the hypothalamic–pituitary level (Ren & Zhu, 2022). An additional important hormone involved in energy metabolism is ghrelin, which increases appetite and body mass (Massadi et al., 2017) and potentially interacts with female sex hormones (Smith et al., 2022). Of the two isoforms of ghrelin present in plasma, acylated and unacylated ghrelin, acylated form is reported to be inversely related to REE (Marzullo et al., 2004; St‐Pierre et al., 2004), while the role of the predominant, unacylated form, remains unclear. These metabolic hormones are suggested to adapt to endurance training in some studies. Baylor and Hackney (2003) proposed that exercise might serve as a signal to conserve energy, leading to a decrease in leptin and thyroid hormones, which has been confirmed in recent trials reporting that higher levels of physical activity are associated with lower thyroid hormone and leptin levels (Fedewa et al., 2018; Klasson et al., 2022). Levels of unacylated ghrelin, by contrast, are shown to increase after chronic exercise training, while acylated ghrelin levels appear to remain the same or decrease (Ouerghi et al., 2021).

The widespread use of combined oral contraceptives (COCs) adds another layer of complexity in studying endurance training adaptations in women. By suppressing endogenous E2 and P4 fluctuations, COCs result in a more stable hormonal profile compared to naturally menstruating women (Elliott‐Sale et al., 2020), which may influence endurance training adaptations. Studies suggest that COC users might have smaller gains in lean mass compared to non‐users (Bozzini et al., 2021; Ihalainen et al., 2019), but research appears to focus primarily on resistance training in athletes and trained women. Therefore, understanding how COC use affects endurance training adaptations in recreationally active women is limited.

There is a demand for rigorously controlled studies that account for hormonal status when investigating the metabolic adaptations to endurance training in women (Elliott‐Sale et al., 2021). Our study addresses this gap by exploring the influence of an 8‐week moderate intensity endurance training (MIET) on REE, body composition, and metabolic hormones in recreationally active healthy women, while carefully controlling for both menstrual cycle and COC cycle phases. This study contributes to metabolic research by aiming to provide a methodological basis for future larger‐scale investigations on endurance training and women's resting metabolism.

## 2 METHODS

### 2.1 Participants

A total of 190 women expressed their interest in the study during 2022–2023, based on advertisements distributed via social media, sports centers, gyms, public spaces, and university mailing lists. Participants were excluded if they did not meet the following criteria: BMI between 19.5 and 35 kg·m^2^, age between 18 and 35 years; non‐smoker; no chronic diseases, medications, or physical disabilities affecting metabolism or physical performance. The training status of most participants was “recreationally active” (Tier 1) according to the Participant Classification Framework by McKay et al. (2021), meaning that they participated in a variety of sports without having a specific commitment or goal. Some of the participants were closer to Tier 0 (McKay et al., 2021), but as none of them were completely sedentary, the term “recreationally active” is used.

Participants were recruited into two groups: naturally menstruating women (NoOC group, *n* = 136) and women using monophasic COC pills (COC group, *n* = 54). The eligibility criterion for a self‐reported menstrual cycle length was 26–35 days over the previous 6 months, while participants in the COC group were required to have taken COCs for at least 3 months before the study. Each participant completed a health questionnaire, which was reviewed by a medical doctor prior to inclusion in the study. The study adhered to the principles of the Declaration of Helsinki and received approval from the Ethics Committee of the University of Jyväskylä (1519/13.00.04/2021). All participants signed an informed consent prior to participation, after receiving a full explanation of the study methods, risks, and benefits.

**FIGURE 1:**
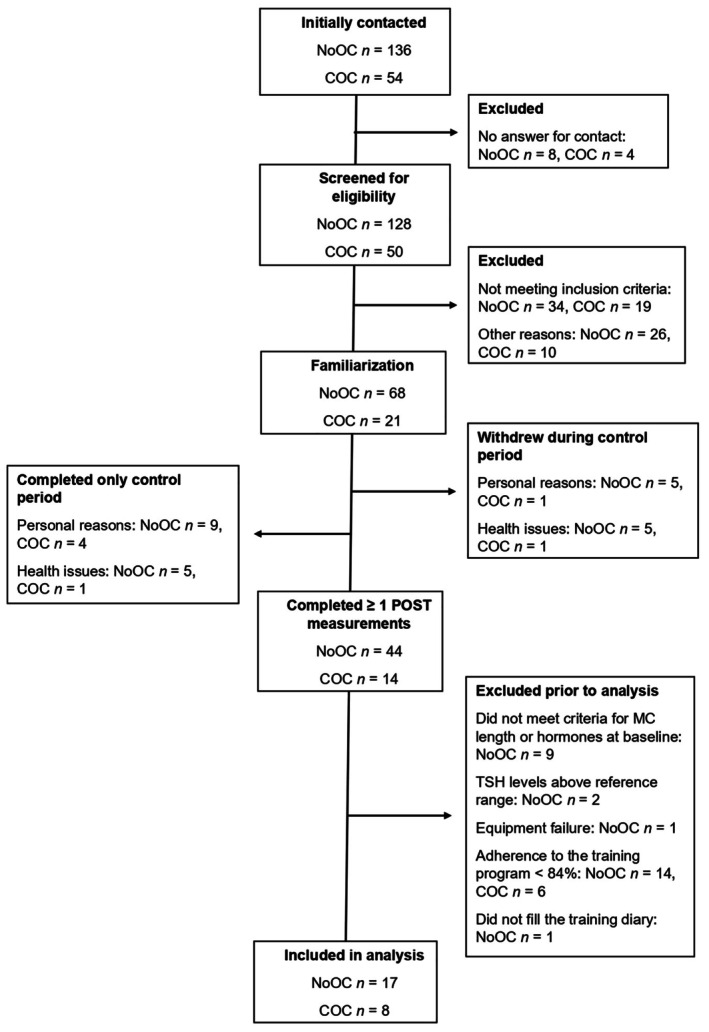
Flowchart illustrating the number of participants at each stage of the study.

Figure 1 illustrates the flowchart from recruitment to analysis. After eligibility screening, 68 NoOC and 21 COC participants proceeded to the familiarization, but 10 NoOC participants and two COC participants withdrew during the control period for personal or health issues before starting the intervention. In addition, 14 NoOC and five COC participants completed only the control period. Of the participants who completed at least one post‐measurement, 27 NoOC participants were excluded retrospectively: P4 levels below 16 nmol·L^−1^ at the luteal phase of the menstrual cycle at baseline (Elliott‐Sale et al., 2021) (*n* = 9), TSH levels above the reference range (*n* = 2), non‐compliance with the training program with adherence <84% (*n* = 14), non‐compliance with filling the training diary (*n* = 1), or equipment failure during the REE measurement (*n* = 1). In the COC group, six participants were excluded due to non‐compliance with the training program. This relatively high dropout rate was possibly due to external factors, such as the ongoing COVID‐19 pandemic. This study uses data from a larger Women's Menstrual Cycle and Endurance Training (NaisQs) study, which had a recruitment goal of at least 10–15 participants per group to achieve a sufficient statistical power. Ultimately, 17 NoOC and 8 COC participants were included in the final analysis. Characteristics of these participants are presented in Table 1. The groups were otherwise similar, but the NoOC group was significantly older (mean difference: 4.2 years, 95% CI: 0.4–7.9, *p* = 0.030).

**TABLE 1:** Baseline characteristics of participants in the NoOC (*n* = 17) and COC groups (*n* = 8), measured at the first measurement (luteal/inactive phases).

	NoOC	COC
Age (years)	31.1 ± 3.9*	26.9 ± 4.8*
Body mass (kg)	66.8 ± 8.3	69.4 ± 7.2
Body fat (%)	28.3 ± 6.5	28.3 ± 5.5
Height (m)	1.66 ± 5.8	1.69 ± 4.9
BMI (kg m^2^)	24.3 ± 3.2	24.3 ± 1.7
$\dot{V}O_{2}$_PEAK_ (mL kg^−1^ min^−1^)	37.6 ± 4.5	39.0 ± 4.2
MC length (d)	28 ± 2	

*Note*: Data are presented as means ± SD. MC length represents the average cycle length during the control period.

Abbreviations: BMI, body mass index; INACT, inactive phase; MC, menstrual cycle; $\dot{V}O_{2}$_PEAK_, peak oxygen uptake.

* *p* ≤ 0.05 between groups.

The COC participants used various COC preparations, including Tasminetta/Yasmin (*n* = 2), Daisynelle (*n* = 1), Dienorette (*n* = 3), Gestinyl (*n* = 1), and Stefaminelle (*n* = 1). All preparations were from 3rd to 4th generation pharmaceutical development. Seven participants took COC pills with the regimen of 21 pill‐taking days (active phase), followed by 7 days of withdrawal (inactive phase), while one participant used COC pills for 24 days followed by 4 days of the inactive phase.

### 2.2 Study design

The study began with a control period, defined as one full menstrual or COC cycle prior to the intervention, during which participants completed daily menstrual diaries. The NoOC group tracked urinary luteinizing hormone and estrone‐3‐glucoronide surges using a Clearblue two‐hormone fertility monitor (Clearblue® Advanced Digital Ovulation, SDP Swiss Precision Diagnostics GmbH (SDP), Geneva, Switzerland). Measurements were completed twice before and after ~8‐week MIET intervention. In the NoOC group, fasting measurements took place at mid‐luteal phase, 4–9 days following an LH surge, and at early‐ to mid‐follicular phase, 1 to 7 days after the onset of menstruation. In addition, the fasting post‐measurements for two NoOC participants were conducted 1 or 2 days before the onset of menstruation due to a scheduling conflict. Based on the retrospective inspection of hormone concentrations, these participants were included in the sample as E2 and P4 had already decreased to the level of early follicular phase (Elliott‐Sale et al., 2021). The COC group measurements were scheduled between Day 2 and 9 of the active phase and between Day 2 and 7 of the inactive phase. These measurements were conducted to record two hormonally distinct phases in both endogenous and exogenous environments.

**FIGURE 2:**
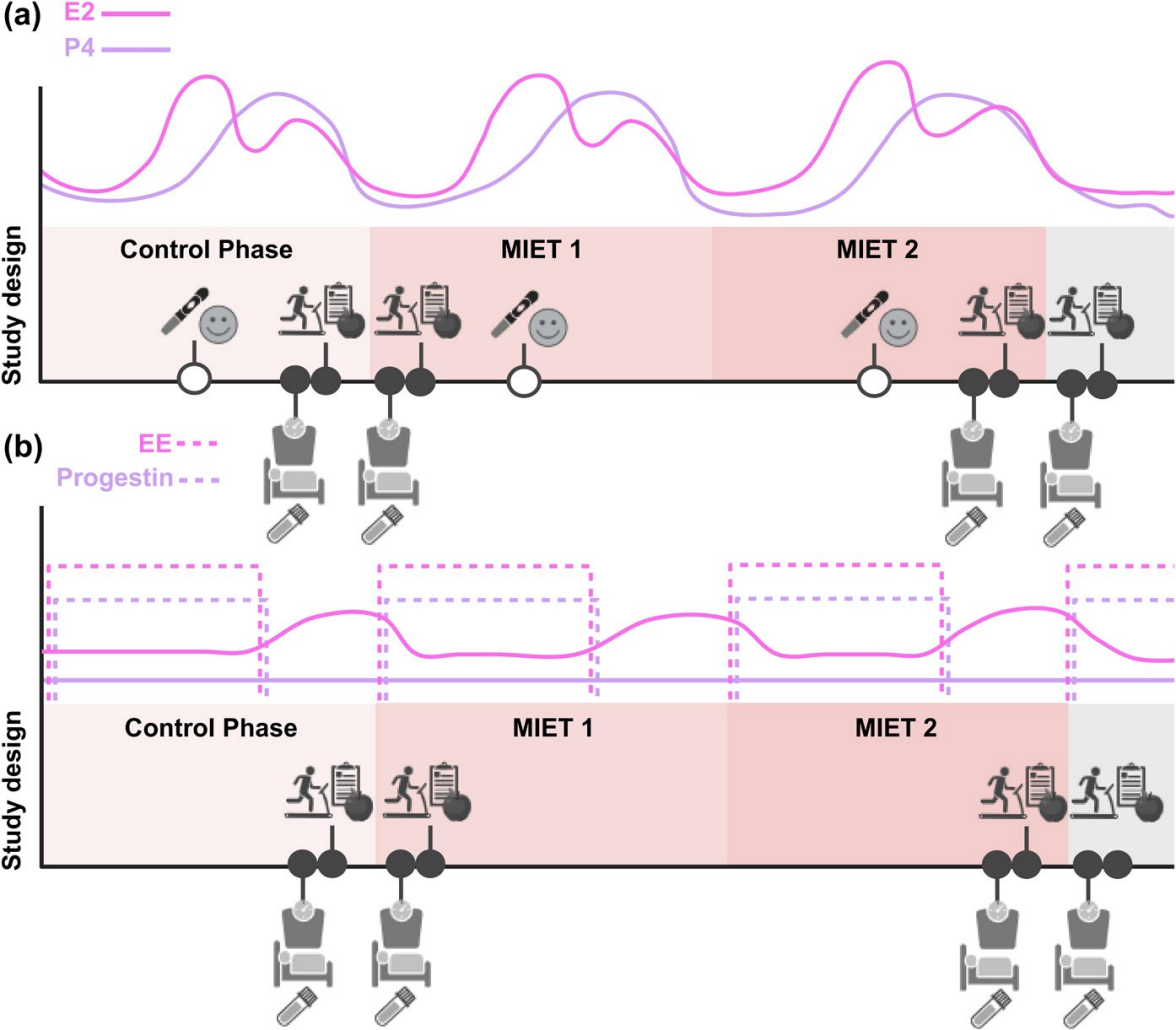
Study design and measurement schedule illustrating hormonal fluctuations across the MC (a) and COC cycle (b). A smile and device icon represents the LH surge test, which preceded the first measurement session. This session included assessment of body composition, REE, and blood sampling. The second measurement involved a $\dot{V}O_{2}$_PEAK_ test, which was followed by a three‐day food diary, beginning the day after the second session. E2: estradiol; P4: progestrone; EE: ethinyl‐E2; MIET: moderate‐intensity endurance training. Created in BioRender, https://BioRender.com/o0n0mmp.

The same protocol was followed across all four measurements. After a 10‐h overnight fast, participants completed REE assessment via indirect calorimetry, body composition measurement using bioimpedance, and blood sampling for hormone analysis. Participants were instructed to refrain from vigorous physical activity and alcohol 24 h before measurements, but they were allowed to drink a glass of water after waking up. The day after, participants completed a peak oxygen uptake ($\dot{V}O_{2}$_PEAK_) test under fed conditions, followed by three days of recording food intake in food diaries. Figure 2 provides a comprehensive overview of the study design.

### 2.3 Intervention

Participants completed two menstrual cycles or two COC cycles (~8‐week) of a MIET program. NaisQs study included a total of a 16‐week endurance training program, consisting of an 8‐week MIET and an 8‐week high‐intensity interval training (HIIT). However, as the HIIT phase suffered from greater dropout, we included only MIET in this analysis. The 8‐week duration was chosen to align with two natural menstrual cycles or COC cycles.

The training program, detailed in Table 2, included running sessions at an intensity of 60%–75% of maximal heart rate (HR_MAX_), as determined by the $\dot{V}O_{2}$_PEAK_ test. Brisk walking was incorporated when necessary to maintain the prescribed heart rate. The total amount of scheduled training was 615 min in the first cycle and 630 min in the second cycle, which was divided into sessions lasting 30–90 min. Training sessions were completed three times per week, except during the first and last weeks, when exercise measurements were performed and adequate rest was required prior to tests. The physiological load of these testing sessions, defined by time spent within the prescribed heart rate range, was considered in the overall training volume. Training sessions were unsupervised due to the COVID‐19 pandemic, but participants recorded each session using a heart rate monitor (Garmin® Venu Series, Garmin Ltd., Taiwan) with a chest strap (Garmin® HRM‐Dual, Garmin Ltd., Taiwan) and filled in the details of their sessions in training diaries.

**TABLE 2:** An 8‐week MIET training program.

Week	Duration (min)	Intensity (%HR_MAX_)
1	30	60–65
	60	60–65
2	45	60–65
	45	70–75
	60	60–65
3	45	60–65
	60	70–75
	75	60–65
4	60	60–65
	60	70–75
	75	60–65
5	45	60–65
	45	70–75
	75	60–65
6	45	60–65
	60	70–75
	90	60–65
7	60	60–65
	60	70–75
	90	60–65
8	60	70–75

*Note*: On Weeks 1 and 8, PFO‐ and VO_2PEAK_ tests were considered in training volume.

Adherence criterion was defined as completing at least ~84% of the scheduled training volume. Adherence was calculated as the percentage of completed training minutes relative to the prescribed goal: % = ((total training minutes/total prescribed minutes) × 100). While 84% of scheduled training results in the training volume of approximately 130 min per week, which is below the 150–200 min recommended for weight gain prevention (Donnelly et al., 2009), this threshold was selected to maintain a sufficient sample size and improve representation of participants who adhered to the study protocol. Moreover, the training program was progressive to accommodate participants who were recreationally active but had not recently completed any structured or performance‐oriented training. The criterion was chosen to balance feasibility with methodological rigor, and variations in training volume were considered when interpreting the results.

Participants were encouraged to adhere to the training sessions as well as they could, along with a consistent time of day for training to minimize the influence of circadian variation (Teo et al., 2011). However, participants were allowed to adjust the schedule of training sessions by a day or two as needed to accommodate work/study and family schedules. Participants were instructed to otherwise maintain their usual daily physical activity and to avoid starting new sports or additional exercise or dietary habits during the study.

### 2.4 Resting metabolism

The canopy method of Vyntus CPX metabolic cart (Vyaire Medical GmbH, Hoechberg, Germany) was used to assess participants' resting metabolism. The gas and flow calibration were conducted according to the manufacturer's instructions before each measurement. Participants rested in a supine position for 10 min in a dim and quiet room before starting the measurement. Oxygen consumption ($\dot{V}O_{2}$) and carbon dioxide production ($\dot{V}{\mathrm{CO}}_{2}$) were recorded for 20 min and averaged at 1‐min intervals. The first 5 min of data and minutes with respiratory exchange ratio (RER) outside the physiological range (0.7–1.0) were discarded before analysis. The criteria for the steady state period were ≤10% coefficient variation (CV) in $\dot{V}O_{2}$ and $\dot{V}{\mathrm{CO}}_{2}$, and ≤5% CV in RER between consecutive minutes (Fullmer et al., 2015). We used the longest, at least 4‐min steady‐state segment for the analysis (Popp et al., 2020). The modified Weir equation was used to calculate the total REE, with an assumption of negligible urinary nitrogen component (Weir, 1949):

$$\mathrm{REE}\;\left(\text{kcal}\cdot d^{-1}\right)=1.44\times \left(3.94\times \dot{V}O_{2}+1.11\times \dot{V}{\mathrm{CO}}_{2}\right)$$

The room temperature ranged from 19.8°C to 24.2°C during measurements (N.B., variable due to seasonal variation). However, a blanket was provided to the participants during the winter (Fullmer et al., 2015).

### 2.5 Body composition

At the first study visit, a wall‐mounted stadiometer was used to measure each participant's height, and a multifrequency bioelectrical impedance device (Inbody 770 body composition analyzer; Biospace Co. Ltd., Seoul, Korea) was used to assess body composition and body mass. Participants wore light clothing, were in a fasted state, and had visited the bathroom before measurements. BMI was calculated as body mass (kg) divided by height squared (m^2^).

### 2.6 $\dot{V}O_{2}$_PEAK_ test

The graded treadmill test was performed on a treadmill (OJK‐1, Telineyhtymä, Kotka, Finland) before and after MIET intervention on a second measurement day. Respiratory gases were measured breath by breath with the Vyntus CPX metabolic cart (Vyaire Medical GmbH, Hoechberg, Germany). Before starting the test, the metabolic cart was calibrated according to the manufacturer's instructions. The participants began with a warm‐up consisting of a 3‐min treadmill walk and dynamic movements. They also performed a countermovement jump test and a maximal bilateral isometric leg press before the $\dot{V}O_{2}$_PEAK_ test, but the results of these tests are not included in this paper. Following the warm‐up, the $\dot{V}O_{2}$_PEAK_ test started at an initial speed of 6 km·h^−1^, with the speed increasing by 1 km·h^−1^ every 3 min until volitional exhaustion. The treadmill was briefly stopped between stages for the fingertip blood lactate samples. The gradient was kept at a constant 0.6° throughout the test. $\dot{V}O_{2}$_PEAK_ was defined as the highest 60‐s rolling average of $\dot{V}O_{2}$, calculated using K‐lab 3.1.25 software (2121.10.01 Aino Health Management Oy, Helsinki). Verbal encouragement was provided to participants throughout the test.

### 2.7 Blood samples

Blood samples were drawn from the antecubital vein into two 6‐mL serum tubes (Vacuette® Tube Greiner Bio‐One GmBH, Kremsmunster, Austria) using standard laboratory procedures. Samples were collected between 6 and 10 am during the fasting measurement. The samples were held at room temperature for 15 min before centrifuging them for 10 min at 2245*g* (Megafuge, 1.0R; Heraeus, Hanau, Germany). The separated serum was stored first at −20°C and then at −80°C for later analysis.

FT3 and female sex hormones (E2, P4) were analyzed using chemiluminescence immunoassays with Immulite®2000 XPi‐analyzator (Siemens Healthcare Diagnostics, New York, NY). Leptin (Biovendor Human LeptinELISA, Brno, Czech Republic), acylated ghrelin (Biovendor Human Ghrelin Acylated Express ELISA), and unacylated ghrelin (Biovendor Human Ghrelin Unacylated Express ELISA) were assessed with an enzyme‐linked immunosorbent assay (ELISA) using Dynex DS 2‐analyzator (Dynex Technologies, Chantilly, VA). The interassay coefficient of variation (CV), determined in our laboratory, and analytical sensitivities for hormones were as follows: fT3 7.6% and 1.5 pmol·L^−1^, E2 8.7% and 55 pmol·L^−1^, and P4 15.5% and 0.3 nmol·L^−1^, leptin 6.7% and 0.2 ng·mL^−1^, acylated ghrelin 6.7% and 5 pg.·mL^−1^, unacylated ghrelin 4.6% and 6 pg.·mL^−1^.

### 2.8 Analysis of training diaries

Participants started to record their training sessions in Excel template‐based training diaries from the first day of the control period and exported the training diaries and the training data from Garmin smartwatches weekly to a data‐secure cloud service (Nextcloud). Training diaries included columns for the date, sport, type of training, duration, distance, rated perceived exertion of session (Robertson, 2004), and the description of training. Training diaries were evaluated by researchers to assess total adherence for training. Any inconsistencies, for example, in session durations and heart rates, were checked from the Garmin training files using the Golden Cheetah open‐source software (Version 3.6) (Golden Cheetah, 2025).

### 2.9 Energy availability calculations

Three‐day food diaries were collected four times during the study to estimate participants' habitual energy intake and minimize the likelihood that any intervention‐induced changes resulted from insufficient energy availability (Siedler et al., 2023). Participants recorded all foods, drinks, and supplements they consumed in a food diary template, estimating portion sizes with household measuring tools or photographs, and were encouraged to complete the diaries as accurately as possible. Furthermore, participants were instructed to maintain their usual diet and refrain from attempting weight loss during the study. The food diaries were analyzed using the Finnish Food Database Fineli (National Institute for Health and Welfare, Helsinki, Finland). Energy availability was calculated by subtracting the exercise energy expenditure (EEE) of intentional training sessions from energy intake, divided by FFM. EEE was calculated using the metabolic equivalent of task (MET) values based on the type, duration, and intensity of activity, according to the Compendium of Ainsworth et al. (Ainsworth et al., 2011). The formula used to calculate EEE is as follows:

$$\mathrm{EEE}=t\times \mathrm{MET}\times \left(\mathrm{REE}/24\right)-\left(\mathrm{REE}/24\right)\times t$$

In the formula, *t* represents the duration of activity, and REE is the measured REE during the fasting measurement.

### 2.10 Statistical analysis

We performed statistical analyses using SPSS Statistics 28 (SPSS Inc., Chicago, IL) and graphed figures with GraphPad Prism (GraphPad Software, Boston, MA). Data are reported as means with standard deviations (SD) or medians with interquartile ranges (IQR), depending on data normality, which was assessed using the Shapiro–Wilk test and visual inspection of histograms and Q‐Q‐plots. Non‐normally distributed variables (E2, P4, leptin, fT3, unacylated ghrelin, acylated ghrelin) were log‐transformed for analyses.

Differences between phases of the menstrual cycle or COC cycle and between the groups at baseline were first analyzed using paired samples *t*‐test or independent samples *t*‐test, or their nonparametric equivalents. Since these results are beyond the scope of this article and have been detailed in a previous publication (Löfberg et al., 2024), they are provided as an additional information in Supplemental Digital Content S1, Table S1. Given that some variables differed between phases, the primary analysis was stratified in two separate phase‐specific comparisons: 1. Pre‐measurements (luteal/active_PRE_) versus post‐measurements (luteal/active_POST_), and 2. pre‐measurements (follicular/inactive_PRE_) versus post‐measurements (follicular/inactive_POST_). The follicular phase was matched with inactive phase due to lower endogenous E2 and P4 during these phases in both groups, while the luteal phase was matched with active phase due to greater hormonal variations between groups (Elliott‐Sale et al., 2020).

Linear mixed models were used as a primary analysis to assess changes over the 8‐week MIET intervention with a model including group, time, and the time × group interaction as fixed effects to investigate changes over time and differences between groups. To avoid overcomplicating the model, we did not include phase as an interaction term and instead conducted two separate comparisons. Given the small sample size, we pooled the NoOC and COC groups together for the analysis of the main effects of time to enhance statistical power. A random intercept for subject ID was included to account for individual variability in results. REE was analyzed both in absolute terms and with FFM and FM as covariates (Heymsfield et al., 2021).

As a secondary, exploratory analysis, we conducted a descriptive, case‐based examination of two participants who demonstrated consistent changes in REE close to or exceeding 10% from both luteal/active_PRE_ to luteal/active_POST_ and from follicular/inactive_PRE_ to follicular/inactive_POST_. The 10% threshold was adapted from Nevin et al. (2024), who justified its use by the evidence that REE tends to vary up to 10% in healthy individuals (Compher et al., 2006). These two cases were examined in more detail to explore whether consistent REE changes were concurrent with alterations in body composition (FFM, FM) or metabolic hormones.

#### 2.10.1 Missing data and outliers

The data set included several missing values, primarily due to illnesses of the participants. Since linear mixed models can accommodate missing data points (Newans et al., 2022), we decided not to completely exclude these cases from our data to maintain the sample size. Details on missing data are provided in Table S2. In addition, one participant completed luteal_POST_ twice due to a lengthened menstrual cycle (43 days). This participant had P4 levels above 16 nmol·L^−1^ in the second luteal_POST_, so data from this measurement were included in the analysis of change from luteal_PRE_ to luteal_POST_. However, a sensitivity analysis was conducted to demonstrate the impact of this participant with a longer cycle on results (Table S3). One outlier in the COC group with REE increase of 331 kcal·d^−1^ (*Z*‐score 2.6) from the inactive_PRE_ to inactive_POST_ was identified based on boxplot inspection. This change exceeded 1.5 times the interquartile range in the pooled sample and was due to an unusually low REE at inactive_PRE_ (1334 kcal·d^−1^) compared with the participant's other measurements. No methodological or technical explanation for the low value was found. Another COC participant (*Z*‐score > −3.0) was classified as unacylated ghrelin outlier based on the standardized pre‐to‐post changes across both phases and boxplot visualization of the changes, even after log transformation. We retained these values in the primary analysis after confirming that they were not due to device or human error but conducted two separate sensitivity analyses excluding these extreme cases (Table S4).

## 3 RESULTS

### 3.1 Training details

Training adherence over the ~8 weeks of MIET was high in both groups: The NoOC group completed 1158 min of MIET training, achieving a 94.6 ± 8.0% adherence rate, while the COC group completed 1154 min of MIET training, with a 94.9 ± 6.0% adherence rate. Furthermore, training adherence was similar between groups, with a mean difference of 0.3% (95% CI: −6.3 to 6.8%, *p* = 0.937).

### 3.2 The effect of MIET on body composition, resting metabolism, and aerobic performance

**FIGURE 3:**
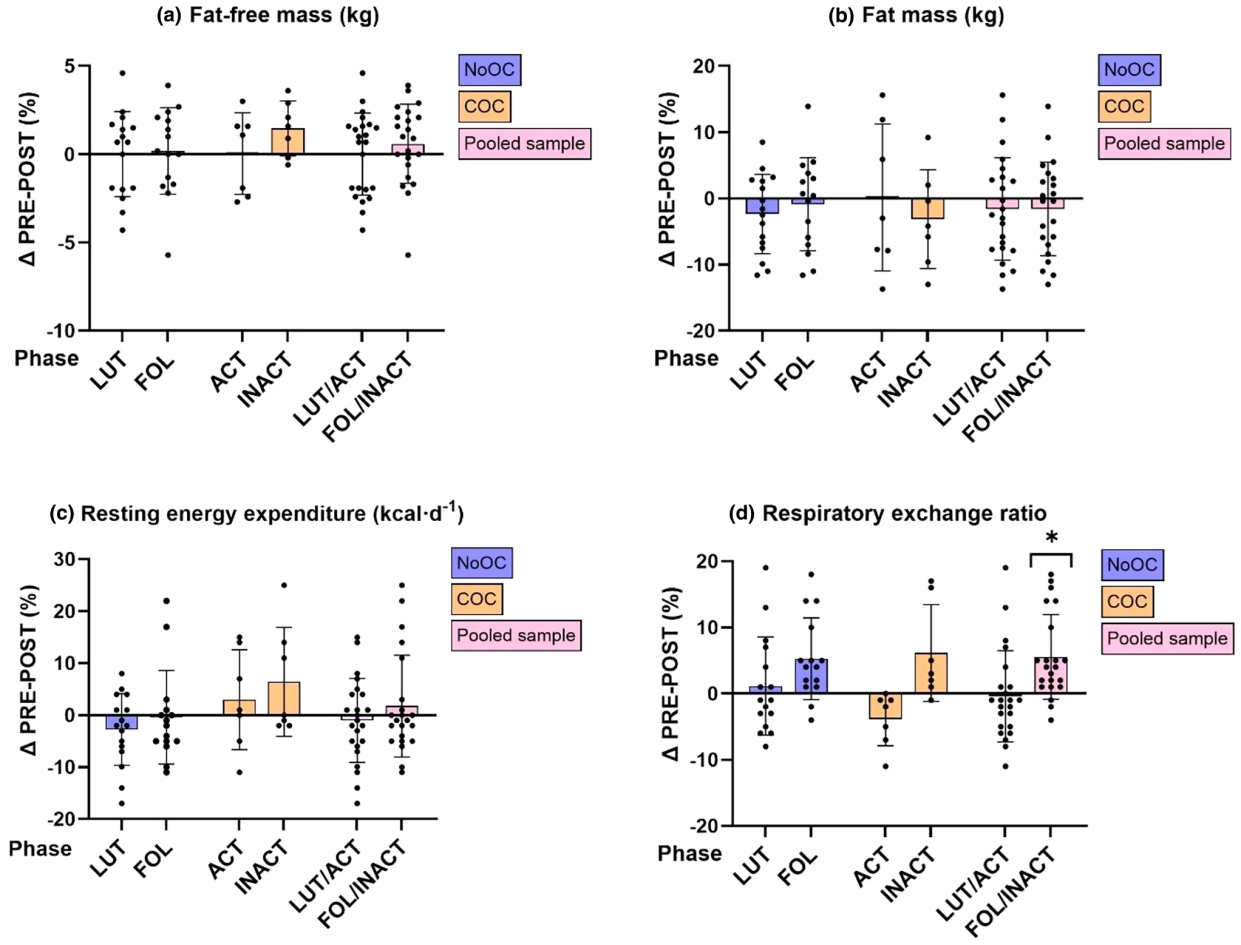
The percentage changes in (a) FFM, (b) FM, (c) REE, and (d) RER from PRE‐ to POST‐MIET. Phase‐specific changes are shown for the luteal phase (*n* = 16) and follicular phase (*n* = 15) (NoOC), active phase (*n* = 7) and inactive phase (*n* = 7) (COC), and luteal/active phases (*n* = 23) and follicular/inactive phases (*n* = 22) (pooled sample). Results are presented as mean and SD, with black dots representing individual values. “*” indicates a main effect of time from pre‐to‐post at follicular/inactive phases *p* ≤ 0.05.

Table 3 presents body composition, resting metabolism, and aerobic performance before and after the 8‐week MIET intervention at luteal/active phase (A) and follicular/inactive phase (B). In the pooled sample, no significant main effect of group, time or interaction effect was observed for FFM, FM, absolute REE (Figure 3A–C) or FFM‐ and FM‐adjusted REE in either phase‐dependent comparison. In both phase comparisons, FFM predicted significantly REE in the pooled sample (luteal/active: *p* = 0.015, follicular/inactive: *p* < 0.001), but FM did not (luteal/active: *p* = 0.460, follicular/inactive: *p* = 0.466). Excluding the REE outlier did not markedly affect the results, either in terms of absolute REE or FFM‐ and FM‐adjusted REE (Table S4).

**TABLE 3:** Body composition, resting metabolism, and aerobic performance before and after the MIET intervention in the NoOC and COC groups. A. Measurements completed at the luteal or active phases. B. Measurements completed at the follicular or inactive phases.

A	NoOC		COC		Pooled sample		*p*‐value		
	LUT_PRE_	LUT_POST_	ACT_PRE_	ACT_POST_	LUT/ACT_PRE_	LUT/ACT_POST_	Group	Time	Time × group
Body composition	*n* = 17	*n* = 16	*n* = 8	*n* = 7	*n* = 25	*n* = 23			
FFM (kg)	47.6 ± 4.0	47.7 ± 4.3	49.9 ± 6.2	49.6 ± 7.3	48.3 ± 4.8	48.3 ± 5.3	0.281	0.896	0.920
FM (kg)	19.3 ± 6.4	19.1 ± 6.4	19.2 ± 4.7	18.9 ± 4.4	19.2 ± 5.8	19.1 ± 5.8	0.966	0.318	0.666
BM (kg)	66.8 ± 8.3	66.8 ± 8.4	69.1 ± 7.1	68.5 ± 7.3	67.6 ± 7.8	67.3 ± 8.0	0.475	0.198	0.432
Resting metabolism	*n* = 17	*n* = 16	*n* = 7	*n* = 6	*n* = 25	*n* = 23			
Absolute REE (kcal·d^−1^)	1452 ± 135	1416 ± 168	1436 ± 131	1462 ± 162	1447 ± 131	1430 ± 164	0.729	1.00	0.139
RER	0.85 ± 0.05	0.86 ± 0.05	0.85 ± 0.02	0.82 ± 0.03	0.85 ± 0.04	0.85 ± 0.04	0.295	0.262	0.121
Aerobic performance	*n* = 17	*n* = 15	*n* = 8	*n* = 7	*n* = 25	*n* = 22			
$\dot{V}O_{2}$_PEAK_ (mL·kg^−1^·min^−1^)	37.6 ± 4.5	38.1 ± 4.1	38.6 ± 3.9	39.7 ± 5.5	37.9 ± 4.3	38.6 ± 4.5*	0.701	0.006*	0.392
$\dot{V}O_{2}$_PEAK_ (L·min^−1^)	2.52 ± 0.31	2.58 ± 0.31	2.71 ± 0.30	2.73 ± 0.39	2.58 ± 0.31	2.63 ± 0.34*	0.253	0.040*	0.246

B	FOL_PRE_	FOL_POST_	INACT_PRE_	INACT_POST_	FOL/INACT_PRE_	FOL/INACT_POST_	Group	Time	Time × group
Body composition	*n* = 14	*n* = 15	*n* = 8	*n* = 7	*n* = 24	*n* = 22			
FFM (kg)	47.5 ± 4.2	47.2 ± 4.6	49.7 ± 6.6	50.8 ± 6.6	48.2 ± 5.1	48.4 ± 5.4	0.213	0.104	0.243
FM (kg)	19.5 ± 6.3	19.6 ± 6.6	19.7 ± 4.3	19.6 ± 4.2	19.5 ± 5.6	19.6 ± 5.9	0.900	0.145	0.407
BM (kg)	66.9 ± 8.5	66.8 ± 9.0	69.4 ± 7.2	70.4 ± 6.3	67.7 ± 8.0	68.0 ± 8.3	0.491	0.951	0.880
Resting metabolism	*n* = 16	*n* = 15	*n* = 8	*n* = 7	*n* = 24	*n* = 22			
Absolute REE (kcal·d^−1^)	1396 ± 174	1381 ± 159	1460 ± 189	1539 ± 154	1417 ± 178	1431 ± 172	0.121	0.236	0.098
RER ($\dot{V}O_{2}$/$\dot{V}{\mathrm{CO}}_{2}$)	0.83 ± 0.04	0.86 ± 0.03	0.83 ± 0.05	0.87 ± 0.07	0.83 ± 0.04	0.87 ± 0.05*	0.789	0.004*	0.827
Aerobic performance	*n* = 14	*n* = 15	*n* = 7	*n* = 7	*n* = 21	*n* = 22			
$\dot{V}O_{2}$_PEAK_ (mL·kg^−1^·min^−1^)	37.4 ± 5.0	38.8 ± 3.7	39.0 ± 4.2	38.3 ± 3.8	37.8 ± 4.7	38.7 ± 3.7	0.650	0.264	0.289
$\dot{V}O_{2}$_PEAK_ (L·min^−1^)	2.47 ± 0.26	2.61 ± 0.26	2.76 ± 0.29	2.72 ± 0.32	2.57 ± 0.29	2.64 ± 0.28	0.119	0.128	0.283

Abbreviations: ACT_POST_, active phase post‐measurements; ACT_PRE_, active phase pre‐measurements; BM, body mass; FFM, fat‐free mass; FM, fat mass; FOL_POST_, follicular phase post‐measurements; FOL_PRE_, follicular phase pre‐measurements; INACT_POST_, inactive phase post‐measurements; INACT_PRE_, inactive phase pre‐measurements; LUT_POST_, luteal phase post‐measurements; LUT_PRE_, luteal phase pre‐measurements; REE, resting energy expenditure; RER, respiratory exchange ratio; $\dot{V}O_{2}$_PEAK_, peak oxygen uptake.

* A main effect of time, *p* ≤ 0.05.

A significant main effect of time was observed for RER, indicating a 5.6% ± 7.5% increase, but this was observed only from follicular/inactive_PRE_ to follicular/inactive_POST_ in the pooled sample (*p* = 0.004) (Figure 3d). In addition, no main effects of group, time or interaction were observed for energy availability in either phase‐dependent comparison. An average energy availability in the pooled sample was 40.3 ± 7.3 kcal·kg FFM^−1^·d^−1^ in pre‐measurements and 40.7 ± 6.9 kcal·kg FFM^−1^·d^−1^ in post‐measurements.

Regarding aerobic performance, absolute $\dot{V}O_{2}$_PEAK_ increased by 2.4 ± 4.1% (*p* = 0.040) and relative $\dot{V}O_{2}$_PEAK_ increased by 2.8 ± 3.8% (*p* = 0.006) in the pooled sample from luteal/active_PRE_ to luteal/active_POST_. From follicular/inactive_PRE_ to follicular/inactive_POST_, absolute $\dot{V}O_{2}$_PEAK_ increased by 3.0 ± 7.4% and relative $\dot{V}O_{2}$_PEAK_ increased 3.1 ± 7.9% in pooled sample (n.s.).

Figure 3c shows large inter‐individual differences in REE changes following the MIET intervention. While several participants had REE changes above 10% in one of the phase comparisons, only two participants, one from the NoOC group and one from the COC group, showed consistent changes in REE across both phases. The NoOC participant showed an average decrease of 10% (9.9%) in REE, accompanied by decreases in FFM (−5%), FM (−7%), and both absolute (−8%) and relative (−9%) $\dot{V}O_{2}$_PEAK_, while fT3 concentrations increased by 24%. By contrast, the COC participant showed an average of 14% increase in REE, with a negligible change in FFM (−1%), an 8% increase in FM, and improvements in both absolute (+6%) and relative (+5%) $\dot{V}O_{2}$_PEAK_, together with a 10% increase in fT3.

### 3.3 The effect of MIET on female sex hormones and metabolic hormones

Table 4 presents hormonal data collected before and after the MIET intervention at luteal/active phase (A) and follicular/inactive phase (B). In the NoOC group, all but one participant with a lengthened menstrual cycle, had P4 levels above 16 nmol·L^−1^ in the luteal phase. A sensitivity analysis excluding this participant did not meaningfully change the results (Table S3).

**TABLE 4:** Female sex hormones and metabolic hormones before and after the MIET intervention in the NoOC and COC groups. A. Measurements completed at the luteal or active phases. B. Measurements completed at the follicular or inactive phases.

A	NoOC		COC		Pooled sample		*p*‐value		
	LUT_PRE_	LUT_POST_	ACT_PRE_	ACT_POST_	LUT/ACT_PRE_	LUT/ACT_POST_	Group	Time	Time × group
Female sex hormones	*n* = 17	*n* = 16	*n* = 8	*n* = 7	*n* = 25	*n* = 23			
E2 (pmol·L^−1^)	503.0 (409.0–734.0)	485.0 (339.8–578.3)	60.6 (26.8–194.3)	53.6 (35.1–136.0)	415.0 (189.5–686.5)	430.0 (136.0–536.0)	<0.001*	0.845	0.839
P4 (nmol·L^−1^)	28.6 (21.1–35.3)	26.0 (21.7–29.7)	1.0 (0.7–1.1)	1.0 (0.8–1.4)	21.4 (1.1–33.7)	23.3 (1.4–27.7)	<0.001*	0.520	0.325
Metabolic hormones	*n* = 17	*n* = 16	*n* = 7	*n* = 7	*n* = 25	*n* = 23			
fT3 (pmol·L^−1^)	4.9 (4.5–5.2)	4.8 (4.5–5.2)	5.6 (4.8–5.7)	5.5 (4.6–6.1)	5.0 (4.7–5.5)	4.9 (4.5–5.4)	0.002*	0.964	0.660
Leptin (ng·mL^−1^)	14.3 (11.8–21.5)	15.9 (9.4–28.6)	15.2 (10.3–32.7)	15.6 (11.0–43.9)	14.6 (11.8–23.8)	15.6 (9.6–29.1)	0.654	0.940	0.786
UnAG (pg·mL^−1^)	316.3 (195.3–476.5)	289.6 (199.5–396.3)	465.0 (289.7–818.1)	269.6 (108.1–498.0)	359.4 (201.7–500.4)	281.1 (195.4–407.0)*	0.643	0.044*	0.104
AG (pg·mL^−1^)	43.5 (37.6–68.9)	52.9 (36.6–110.0)	58.5 (41.5–340.7)	75.6 (38.8–231.2)	43.5 (39.2–94.5)	54.8 (37.9–123.1)	0.640	0.971	0.617

B	FOL_PRE_	FOL_POST_	INACT_PRE_	INACT_POST_	FOL/INACT_PRE_	FOL/INACT_POST_	Group	Time	Time × Group
Female sex hormones	*n* = 16	*n* = 14	*n* = 8	*n* = 7	*n* = 24	*n* = 21			
E2 (pmol·L^−1^)	114.0 (89.7–163.0)	109.1 (80.5–153.8)	38.6 (24.9–151.3)	65.3 (32.6–125.0)	103.0 (42.6–163.0)	98.4 (62.6–133.5)	0.004*	0.522	0.416
P4 (nmol·L^−1^)	1.1 (0.6–1.6)	1.5 (0.9–2.1)	0.7 (0.4–1.2)	0.7 (0.4–1.4)	0.9 (0.5–1.4)	1.2 (0.8–1.8)	0.013*	0.856	0.293
Metabolic hormones	*n* = 16	*n* = 14	*n* = 8	*n* = 7	*n* = 24	*n* = 21			
fT3 (pmol·L^−1^)	4.7 (3.9–5.3)	5.0 (4.7–5.2)	5.9 (5.7–6.3)	6.2 (5.8–6.3)	5.2 (4.2–5.8)	5.2 (4.9–6.1)	<0.001*	0.230	0.742
Leptin (ng·mL^−1^)	11.7 (8.2–18.0)	12.6 (7.1–24.9)	13.5 (10.5–27.3)	19.6 (10.4–33.6)	12.8 (8.5–18.0)	13.2 (8.9–27.5)	0.287	0.107	0.527
UnAG (pg·mL^−1^)	336.9 (295.8–467.8)	229.7 (162.3–524.3)	428.6 (270.5–847.5)	249.0 (185.4–454.1)	346.5 (295.8–492.4)	245.3 (173.7–488.5)	0.692	0.031*	0.502
AG (pg·mL^−1^)	46.8 (37.4–77.2)	57.7 (44.1–163.7)	64.2 (41.2–287.6)	66.4 (36.6–313.5)	48.8 (38.1–86.9)	61.3 (42.9–178.2)	0.712	0.872	0.328

Abbreviations: ACT_POST_, active phase post‐measurements; ACT_PRE_, active phase pre‐measurements; AG, acylated ghrelin; E2, estradiol; FOL_POST_, follicular phase post‐measurements; FOL_PRE_, follicular phase pre‐measurements; fT3, free triiodothyronine; INACT_POST_, inactive phase post‐measurements; INACT_PRE_, inactive phase pre‐measurements; LUT_POST_, luteal phase post‐measurements; LUT_PRE_, luteal phase pre‐measurements; P4, progesterone; unAG, unacylated ghrelin.

* A main effect of time or group, *p* ≤ 0.05.

Expected differences between the groups were observed for E2 and P4, with significantly higher concentrations in the NoOC group compared to the COC group (luteal/active: E2, *p* < 0.001, P4, *p* < 0.001; follicular/inactive: E2, *p* = 0.004, P4, *p* = 0.013). A group effect for fT3 was observed in both phases (luteal/active: *p* = 0.002, follicular/inactive *p* < 0.001), showing overall higher concentrations in the COC group compared to the NoOC group.

A main effect of time was observed for uncylated ghrelin from luteal/active_PRE_ to luteal/active_POST_ in the pooled sample, indicating a decrease (*p* = 0.044) and from follicular/inactive_PRE_ to follicular/inactive_POST_ (*p* = 0.031). However, after excluding one unacylated ghrelin outlier, the main effect of time was not significant anymore (luteal/active: *p* = 0.195, follicular/inactive: *p* = 0.114) (Table S4).

## 4 DISCUSSION

The present study found no evidence suggesting a major influence of an 8‐week MIET intervention on REE, body composition, and metabolic hormones in recreationally active naturally menstruating and COC using women. However, we observed large individual variability in outcome variables and slight improvements in $\dot{V}O_{2}$_PEAK_ in the pooled sample. Unfortunately, recruitment challenges resulted in a relatively small sample size, particularly in the COC group, limiting the statistical power to detect potential differences between the groups. As such, rather than drawing definitive conclusions about group differences, this study provides preliminary information on potential hormonal and metabolic adaptations in the pooled sample combining naturally menstruating and COC using women, representing real‐life conditions.

### 4.1 Influence of MIET on REE and body composition in the pooled sample

Our finding of a stable REE after MIET intervention is consistent with the latest review by MacKenzie‐Shalders et al. (2020), which reported that endurance training does not have a clear influence on REE. The review also outlined that several studies lacked sufficient body composition data and reporting of methodological details. However, even well‐controlled, large‐scale studies failed to find changes in REE independent of changes in body composition following a 20‐week (Martin et al., 2019) or a 10‐month of supervised training interventions (Willis et al., 2014) in mixed sex, overweight or obese untrained participants. However, exceptions exist, as Potteiger et al. (2008) reported 129 ± 154 kcal·d^−1^ higher REE after 16‐month of moderate intensity aerobic endurance training in moderately obese or overweight untrained women. In contrast, Byrne and Wilmore (2001) observed opposite results, with decreased REE (−53 kcal·d^−1^) in obese naturally menstruating women following a 20‐week resistance and aerobic endurance training intervention (Byrne & Wilmore, 2001). The reason for the decreased REE in the study of Byrne and Wilmore (2001) is unclear. The authors speculated that it could be due to heat acclimation, as participants completed their aerobic training outdoors during the summer in high temperature and humidity. However, their study is not entirely comparable to ours due to the resistance training component involved in their intervention.

In addition to varying protocols with respect to mode, frequency, intensity, and duration of intervention among previous studies, one crucial factor contributing to the divergent results could be the difficulty in precisely timing REE measurements after physical activity, as prolonged excess post‐exercise oxygen consumption could last for up to 36–48 h (Speakman & Selman, 2003). It is generally recommended that participants avoid moderate to vigorous physical activity for 12–48 h before REE measurement, while 90‐min resistance training extends the recommended recovery period to at least 48 h (Fullmer et al., 2015), based on one study reporting elevated REE 48 h after a single resistance training session in older males (Williamson & Kirwan, 1997). However, Binzen et al. (2001) demonstrated a significant increase in excess post‐exercise oxygen consumption in naturally menstruating women of similar age to our sample, which returned to baseline within 2 h after a resistance training session. Given these findings, our instruction to avoid vigorous physical activity for 24 h prior to measurements should have been sufficient to minimize the potential confounding effect of acute exercise in this population.

Another potential limitation in previous studies complicating the interpretation of differences in REE adaptations may be the adjusting method of REE to body composition. That is, the approach of ratio scaling (REE divided by FFM or body mass) used by some studies (Lee et al., 2009; Potteiger et al., 2008; Scharhag‐Rosenberger et al., 2010) assumes a proportional relationship between REE and body mass with a zero intercept, which does not represent tissue‐specific metabolic rates and may lead to misleading conclusions (Müller et al., 2021; Tanner, 1949). In our study, we used a regression‐based approach to adjust REE for body composition, which has been demonstrated to provide a more accurate adjustment (Müller et al., 2021), thereby avoiding the limitations associated with ratio scaling.

In the present study, FFM was a significant predictor of REE across both menstrual cycle and COC phases, in agreement with the well‐known association between FFM and REE (Heymsfield et al., 2021). Given that FFM remained stable across the MIET intervention, it is plausible that this explains the lack of change in REE at a group‐level. The lack of change in FFM was a somewhat expected finding, as endurance training interventions have typically not been shown to elicit muscle hypertrophy (Ross et al., 2024). However, some researchers have changed the paradigm to recognize the anabolic potential of long‐term endurance training, particularly in physically inactive individuals after cycling interventions (Konopka & Harber, 2014). In the present study, participants were recreationally active, and the MIET was completed primarily by running, which may explain the lack of meaningful FFM gains. In addition, FM remained consistent on group level, probably due to the absence of dietary control, as participants were instructed to follow their habitual eating patterns to avoid the confounding effect of energy deficiency on body composition and REE (Schwartz & Doucet, 2010). According to the meta‐analysis by Shaw et al. (2006), weight loss achieved through exercise alone is marginal (0.5–4 kg) in long‐term interventions (≥3 months), compared to interventions including concurrent dietary intervention. The unchanged energy availability from pre‐to‐post in the present study further supports the observation of stable body composition, although it should be noted that energy availability calculation is based on self‐reported food and training diaries and as such is prone to errors (Burke et al., 2018).

### 4.2 Influence of MIET on metabolic hormones in the pooled sample

Our study did not find evidence of consistent changes in fT3 or leptin following the MIET intervention, which disagrees with some previous studies reporting reductions in fT3 and leptin in women after exercise intervention. Boyden et al. (1982) observed decreased total T3 concentrations in regularly jogging female participants after a progressive running program. However, the total T3 used in the study of Boyden et al. (1982) is not equivalent to the measurement of fT3, which is considered the biologically active form of T3 (Welsh & Soldin, 2016). Correspondingly, Baylor and Hackney (2003) classified female collegiate athletes as responders and non‐responders based on fT3 decrease after a 20‐week training program, with leptin levels following ft3 levels in responders (Baylor & Hackney, 2003). In contrast, Bozzini et al. (2021) investigated female soccer players over a 16‐week competitive season and reported a gradual increase in leptin levels, while fT3 initially increased until week 12 before declining through week 15. The reason for the observed increases in fT3 and leptin is unknown, but the authors proposed that they might reflect changes in energy balance (Bozzini et al., 2021). However, the training status of participants as well as the duration and type of training in these studies differed vastly from our present study, limiting the comparability.

Studies examining fT3 changes after MIET intervention in untrained or recreationally active women are sparse, but FM‐independent leptin decreases (Hickey et al., 1997) and no changes in leptin (Arikawa et al., 2011) following aerobic exercise intervention have been reported in untrained women. The lack of change in leptin is most likely explained by stable FM, which is the primary source of leptin (Karim & Afiq, 2011). Conversely, a proposed mechanism for both leptin and fT3 decrease is downregulation of the hypothalamic–pituitary‐thyroid axis as a protective response to exercise‐induced stress, in order to conserve energy (Steinacker et al., 2005). It must be noted that some of the discrepancies between discussed studies might be explained by methodological differences, as some have used radioimmunoassays (Baylor & Hackney, 2003; Boyden et al., 1982; Hickey et al., 1997) and some automated immunoassay techniques (Arikawa et al., 2011; Bozzini et al., 2021), including the present one.

In our study, acylated ghrelin remained stable in the pooled sample, while significant decrease in unacylated ghrelin was driven by one COC participant with a marked and consistent decrease in uncylated ghrelin following the MIET. Both unacylated ghrelin and acylated ghrelin have been shown to decrease with obesity and represent positive energy balance (Wang et al., 2022), but as this non‐obese participant had stable body composition and acylated ghrelin levels, our data does not allow us to track the exact physiological mechanism behind this outlier. Previous studies exploring the adaptations in unacylated ghrelin and acylated ghrelin to aerobic exercise training in untrained or recreationally active women are sparse and conflicting. In two studies, involving either middle‐aged women (Ueda et al., 2013) or adult men and women (Martins et al., 2010), 12‐week aerobic exercise increased acylated ghrelin levels in parallel with body fat reductions. Another study comparing moderate‐dose and low‐dose 4‐month aerobic exercise intervention in older women reported decreased acylated ghrelin levels in the moderate‐dose group, and adjustment for body mass and FM changes did not affect this result (Bowyer et al., 2019). Acylated ghrelin represents ~10% of total plasma ghrelin and undergoes a post‐translational addition of fatty acid, which allows it to activate the growth hormone secretagogue receptor and exert primary metabolic actions of ghrelin, while the metabolic effects of unacylated ghrelin might be different (Cappellari & Barazzoni, 2019). Unacylated ghrelin was shown to increase in association with reduced FM among a large sample of men after 6‐month military training (Cederberg et al., 2011), and this was observed in obese women as well, but independently of FM loss, in a very similar design to ours (8‐week aerobic endurance exercise intervention) (Mirzaei et al., 2009). However, overall evidence suggests that changes in acylated and unacylated ghrelin after aerobic exercise appear to be tightly linked to changes in body mass (King et al., 2013).

### 4.3 Phase‐specific explorations, individual variability, and group differences

To our knowledge, this is the first study investigating resting metabolism across menstrual and hormonal contraceptive phases before and after an endurance training intervention. We found no evidence of phase‐specific training adaptations in REE. However, our approach of combining two hormonally distinct groups might dilute potential phase‐related changes, as REE among COC users appears to remain stable along with sex hormone levels (Duhita et al., 2017). While it has been suggested that REE increases slightly from the early follicular to mid‐luteal phase (Benton et al., 2020; Löfberg et al., 2024), the typical day‐to‐day variability up to 10% in REE (Compher et al., 2006) might override the potential effects of both menstrual cycle and training.

In the present study, we found substantial intra‐individual variation in REE across measurements, despite the standardized conditions and adherence to the recommended analysis protocol (Fullmer et al., 2015). One COC participant exhibited a 25% increase in REE between pre‐ and post‐inactive phase measurements due to the low REE value at pre‐measurement. A similar pattern was not observed when comparing pre‐ and post‐active phase REE data in this participant. The exceptionally low REE could be explained by the summed effects of natural biological variability and subtle methodological bias, such as acute energy deficiency and ventilatory changes (McClave et al., 2003; Siedler et al., 2023). It has been reported that an energy restriction period of as short as 4 days can decrease REE up to 13% (Kouda et al., 2006). Importantly, our finding illustrates how challenging it is to distinguish true biological variability from the unexplained noise in REE measurements.

Only two individuals demonstrated consistent REE changes (≥10%) across both phases. The REE decrease in one NoOC participant may be due to the loss of both FFM, considering the linear relationship between REE and FFM (Müller et al., 2018). In contrast, the COC participant showed an increase in REE with stable FFM and slightly increased FM (8%). FM contributes to REE variance less due to its lower metabolic rate (Wang et al., 2022), which is a pattern we observed as well, so it is unlikely that FM increase alone explains the REE change. Both participants had a marked increase in fT3, which is unexpected particularly for the NoOC participant, considering the role of fT3 as a key hormone driving REE (Yavuz et al., 2019). While being highly speculative and explorative, consistent changes across both phases might reflect true physiological adaptations instead of random variability. Although causal conclusions cannot be drawn, these two cases might highlight the potential value of individual‐level analyses complementing group‐level statistics in studies showing high physiological variability.

We observed high inter‐individual variability in the body composition changes. The reason why some women gained, and others lost FFM or FM despite the similar training volume, cannot be directly explained by the gathered data. However, it is likely that individual factors, such as those related to diet and hydration status, have played a role. As participants completed a three‐day food diary only at four points of the study (before and after the intervention), we do not have data on energy intake during the intervention. It is possible that participants' energy intake fluctuated during the intervention despite instructions to maintain a habitual diet and physical activity (previous regular activities) throughout the intervention, as the aim was to investigate how added MIET would affect the outcomes. While the current consensus suggests that controlling for menstrual and COC cycle phase is not needed in body composition assessments performed by bioelectrical impedance (Rael et al., 2020; Schröder et al., 2024), changes in fluid balance can still affect results as water is a central component of FFM (Son et al., 2025). Dual energy X‐ray absorptiometry, considered the gold standard in body composition assessment (Kim, 2024), would have been a more accurate method for assessing body composition. However, research regulations in Finland restrict its frequent use, as the method involves radiation exposure.

In the pooled sample, RER was higher at follicular/inactive_POST_ compared to follicular/inactive_PRE_, but not at luteal/active_PRE_ compared to luteal/active_POST_. In addition, a significant main effect of time, indicating increases in both absolute and relative $\dot{V}O_{2}$_PEAK_, was observed from luteal/active_PRE_ to luteal/active_POST_, but not from follicular/inactive_PRE_ to follicular/inactive_POST_. The inconsistent changes in RER could be explained by diet‐related factors on the days preceding the measurements rather than hormonal shifts, as fasting RER has been shown to increase by approximately 0.10 in response to a 30% increase in carbohydrate intake (Miles‐Chan et al., 2015). Due to the logistical challenges, we did not monitor diet on the days preceding the measurements. Furthermore, the meta‐analysis and systematic review reported stable RER levels at rest across the menstrual and COC cycle (D'Souza et al., 2023). However, definitive conclusions cannot be drawn, as the studies included in the review were rated as providing low certainty of evidence. Our study does not offer a clear physiological explanation for the differential response in $\dot{V}O_{2}$_PEAK_ across phases. They likely reflect, at least partly, variability in personal factors, such as acute changes in nutrition or hydration (Jeukendrup, 2014), sleep (Lopes et al., 2023), or motivation (McCormick et al., 2015).

Metabolic adaptations to MIET did not appear to be influenced by COC use. While differences in REE following the exercise intervention between COC users and non‐users have not been previously examined, some studies with different designs have explored metabolic hormones and body composition in these two groups. Bozzini et al. (2021) reported substantial differences between groups in body composition among female soccer players over a 16‐week competitive season. Women using unspecified oral contraceptives experienced smaller gains in FFM with no change in body fat, whereas naturally menstruating women had greater FFM increases along with reductions in body fat (Bozzini et al., 2021). Similar findings were reported by Ihalainen et al. (2019) following a 10‐week high‐intensity combined strength and endurance training, potentially due to elevated inflammatory markers in COC users.

It has been suggested that the estrogenic component of COCs increases the levels of thyroxine‐binding globulin, a protein that carries T3 in the bloodstream (Schussler, 2000), leading to the increased total T3 concentrations in COC users (Torre et al., 2020). However, the impact of COC on fT3 is unclear, as most studies have suggested it to remain stable or decrease slightly due to the increased serum binding (Sänger et al., 2008; Wiegratz et al., 2003). Contrary to these findings, we found higher concentrations of fT3 in the COC group compared to the NoOC group. This indicates that the influence of COC use on thyroid metabolism may not be limited to the binding dynamics. Moreover, the artificial suppression of the hypothalamic–pituitary–ovarian axis by exogenous hormones may create different metabolic states with elevated fT3 levels, which has not been widely addressed in prior literature. Therefore, further research is warranted to explore the physiological relevance of increased fT3 in COC users.

### 4.4 Strengths and limitations

Despite the novel study design and rigorous control over menstrual cycle and COC phases, as well as standardized REE measurement procedures, our study had several limitations. Since our study did not include a non‐exercising control group, it remains uncertain whether the absence of significant changes in the main outcomes reflects a true lack of intervention effect or other potential factors, such as natural variation. Second, a control period of one menstrual cycle may have been insufficient to account for within‐participant variation in menstrual cycle characteristics (Elliott‐Sale et al., 2021). On the other hand, over a third of natural menstrual cycles are suggested to be anovulatory (Prior et al., 2015), which is why a longer control period does not necessarily guarantee a stable baseline. Furthermore, a relatively small sample size and imbalanced group distribution impaired statistical power, which may have limited our ability to detect subtle differences in outcomes. The sample size also prevented us from using more advanced statistical methods, such as structural equation modeling, to examine potential mediators of REE changes. The length of intervention was also relatively short. Therefore, our approach was exploratory, and findings should be interpreted as such.

A major strength in the present study was the repeated measurements in both follicular/inactive phases and luteal/active phases, which allowed us to gain insight into potential hormonal influences on exercise‐induced metabolic adaptations. However, as we chose not to include a time × group × phase interaction in the linear mixed models to reduce the complexity of interpreting the three‐way interaction in such a small sample, we cannot draw sound conclusions about the influence of hormonal phase on metabolic adaptations. Finally, given the lack of significant time × group interactions, our discussion focused on the main effects of time in the pooled sample. While this approach provided results that reflect the real‐life variability in hormonal statuses among women, it compromises the interpretation of differing hormonal profiles between naturally menstruating and COC‐using women.

## 5 CONCLUSIONS

The 8‐week MIET did not result in detectable adaptations in REE, body composition, or metabolic hormones among naturally menstruating and COC‐using women. We found no evidence of hormonal status influencing results, despite overall higher fT3 concentrations in COC‐using women. However, high individual variations in adaptations suggest that some individuals may experience substantial metabolic changes even when mean effects are small. From a practical perspective, longer or more intensive interventions, perhaps in combination with nutritional strategies and resistance training, may be required to achieve measurable metabolic adaptations in recreationally active women.

## AUTHOR CONTRIBUTIONS

R.S.M., J.K.I., H.K., A.C.H., I.E.L., and V.M.S. conceptualized the study. Methodology was developed by R.S.M., J.K.I., I.E.L., and V.M.S. Investigation was conducted by I.E.L., V.M.S., and A.K. Data curation and formal analysis were performed by I.E.L., V.M.S., and R.S.M. Resources were provided by R.S.M. and J.K.I. The original draft was written by I.E.L. and R.S.M., with review and editing by A.K., V.M.S., J.K.I., H.K., and A.C.H. The work was supervised by R.S.M., J.K.I., H.K., and A.C.H. Funding acquisition and project administration were led by R.S.M., with support from V.M.S. and I.E.L.

## FUNDING INFORMATION

The NaisQs study was financially supported by the Finnish Ministry of Education and Culture (Grant No. OKM/21/626/2021, OKM/101/626/2021, OKM/82/626/2022) and Firsbeat Analytics Oy. Garmin Venu 2S smartwatches and Garmin HRM‐dual heart rate monitors were provided by Firstbeat Analytics Oy. Additional funding for blood analyses was provided by Suomen Urheilututkimussäätiö.

## CONFLICT OF INTEREST STATEMENT

The authors have no conflict of interest to declare.

## Supporting information


Data S1.

## Data Availability

The data analyzed during the current study are available from the corresponding authors upon reasonable request.
