# Supplementary material for: Influence of 8‐week endurance training on resting energy expenditure and body composition in women
Source: Physiol Rep. 2025 Sep 30;13(19):e70591. doi: 10.14814/phy2.70591 (PMC12484297; doi:10.14814/phy2.70591)
Supplement: Supplementary file 1 — Data S1. [file PHY2-13-e70591-s001.docx]

**Supplemental Digital Content**

**Page 1–2, Supplemental Table 1.** Descriptive statistics and differences between menstrual or COC cycle phases and groups for pre-measurements in NoOC and COC groups.

**Page 3–4. Supplemental Table 2.** Summary of missing data and reasons across all measurements in NoOC and COC groups.

**Page 5, Supplemental Table 3.** Body composition, resting metabolism, aerobic performance, and hormones before and after the MIET intervention in the NoOC group and pooled sample after the sensitivity analysis excluding a participant with lengthened menstrual cycle, measurements completed at luteal or active phases.

**Page 6, Supplemental Table 4.** Results after sensitivity analysis for REE from A) follicular/inactive_PRE_ to follicular/inactive_POST,_ in the COC group and the pooled sample, excluding one participant based on boxplot analysis_,_ and for unacylated ghrelin from B) luteal/active_PRE_ to luteal/active_POST_ and C) follicular/inactive_PRE_ to follicular/inactive_POST_ in the COC group and the pooled sample, excluding one participant with Z-score of ±3.

**Within group and between group differences before MIET intervention**

Before the MIET intervention, the NoOC group demonstrated higher E2 and P4 levels in the luteal phase compared to the follicular, as expected (E2: *p* < 0.001; P4: *p* < 0.001). Leptin levels were also higher in the luteal compared to the follicular (*p* = 0.002). Energy availability was 6.1 kcal·kg FFM^−1^·d^−1^ higher in the luteal compared to the follicular (95% CI: 0.5 to 11.6 kcal·kg FFM^−1^·d^−1^, *p* = 0.036). Other variables remained stable between phases. In the COC group, body mass was 0.2 kg higher in the inactive than the active (95% CI: 0.1 to 0.5, *p* = 0.046). In addition, fT3 was higher in the inactive compared to the active (*p* = 0.012).

Between-group comparisons showed that the NoOC group had higher E2 and P4 levels in the luteal compared to the active (E2: *p* = 0.003; P4: *p* < 0.001) and higher E2 levels in the follicular compared to the inactive (*p* = 0.037), as expected. In contrast, the COC group exhibited higher fT3 levels in the inactive than the NoOC group in the follicular (*p* < 0.001). Absolute V̇O_2PEAK_ was slightly higher in the inactive of the COC group than the follicular of the NoOC group (mean difference 0.3 L∙min^-1^, 95% CI: 0.0 to 0.5 L∙min^-1^, *p* = 0.035).

**Supplemental Table 1.** Within-group and between-group differences in body composition, resting metabolism, aerobic performance, hormones and energy availability at two menstrual cycle or COC phases before MIET intervention in NoOC and COC groups. Results are presented as means ± SD or medians (IQR).

|  | **NoOC** | | **COC** | |
| --- | --- | --- | --- | --- |
|  | **LUT_PRE_** | **FOL_PRE_** | **ACT_PRE_** | **INACT_PRE_** |
| **Body composition** | *n* = 17 | *n* = 16 | *n* = 8 | *n* = 8 |
| FM (kg) | 47.6 ± 4.0 | 47.5 ± 4.2 | 49.9 ± 6.2 | 49.7 ± 6.6 |
| FFM (kg) | 19.3 ± 6.4 | 19.5 ± 6.3 | 19.2 ± 4.7 | 19.7 ± 4.3 |
| BM (kg) | 66.8 ± 8.3 | 66.9 ± 8.5 | **69.1 ± 7.1*** | **69.4 ± 7.2*** |
| **Resting metabolism** | *n* = 17 | *n* = 16 | *n* = 7 | *n* = 8 |
| Absolute REE (kcal·d^-1^) | 1452 ± 135 | 1396 ± 174 | 1436 ± 131 | 1460 ± 189 |
| RER (V̇O_2_/V̇CO_2_) | 0.85 ± 0.05 | 0.83 ± 0.04 | 0.85 ± 0.02 | 0.83 ± 0.05 |
| **Aerobic performance** | *n* = 17 | *n* = 14 | *n* = 8 | *n* = 8 |
| V̇O_2PEAK_  (mL∙kg^-1^∙min^-1^) | 37.6 ± 4.5 | 37.4 ± 5.0 | 38.6 ± 3.9 | 39.0 ± 4.2 |
| V̇O_2PEAK_ (L∙min^-1^) | 2.52 ± 0.31 | **2.47 ± 0.26†** | 2.71 ± 0.30 | **2.76 ± 0.29†** |
| **Female sex hormones** | *n* = 17 | *n* = 16 | *n* = 8 | *n* = 8 |
| E2 (pmol·L^−1^) | **503.0 (409.0–734.0)*†** | **114.0 (89.7–163.0)*†** | **60.6 (26.8–194.3)†** | **38.6 (24.9–151.3)†** |
| P4 (nmol·L^−1^) | **28.6 (21.1–35.3)*** | **1.1 (0.6–1.6)*** | **1.0 (0.7–1.1)†** | 0.7 (0.4–1.2) |
| **Metabolic hormones** | *n* = 17 | *n* = 16 | *n* = 7 | *n* = 8 |
| fT3 (pmol·L^−1^) | 4.9 (4.5–5.2) | **4.7 (3.9–5.3)†** | **5.6 (4.8–5.7)*** | **5.9 (5.7–6.3)*†** |
| Leptin (ng·mL^−1^) | **14.3 (11.8–21.5)*** | **11.7 (8.2–18.0)*** | 15.2 (10.3–32.7) | 13.5 (10.5–27.3) |
| UnAG (pg·mL^−1^) | 316.3 (195.3–476.5) | 336.9 (295.8–467.8) | 465.0 (289.7–818.1) | 428.6 (270.5–847.5) |
| AG (pg·mL^−1^) | 43.5 (37.6–68.9) | 46.8 (37.4–77.2) | 58.5 (41.5–340.7) | 64.2 (41.2–287.6) |
| **Dietary measures** | *n* = 16 | *n* = 15 | *n* = 7 | *n* = 7 |
| EA (kcal·kg FFM^-1^·d^-1^) | **43.7 ± 9.8*** | **39.9 ± 6.6*** | 38.7 ± 9.7 | 39.5 ± 6.2 |

LUT_PRE_: luteal phase pre-measurements; FOL_PRE_: follicular phase pre-measurements; ACT_PRE_: active phase pre-measurements; INACT_PRE_: inactive phase pre-measurements; FFM: fat-free mass; FM: fat mass; BM: body mass; REE: resting energy expenditure; RER: respiratory exchange ratio: V̇O_2PEAK_: peak oxygen uptake; E2: estradiol; P4: progesterone; fT3: triiodothyronine; UnAG: unacylated ghrelin; AG: acylated ghrelin; EA: energy availability. ‘*’ indicates a significant difference between phases (*p* ≤ 0.05)

‘†’ indicates a significant difference between groups (*p* ≤ 0.05)

**Missing data**

**Supplemental Table 2**. Summary of missing data and reasons across all measurements in NoOC and COC groups.

| **Variable** |  | | | |  | | | | **Reason** |
| --- | --- | --- | --- | --- | --- | --- | --- | --- | --- |
|  | **LUT_PRE_** | **LUT_POST_** | **FOL_PRE_** | **FOL_POST_** | **ACT_PRE_** | **ACT_POST_** | **INACT_PRE_** | **INACT_POST_** |  |
| FFM | 0 | 1 | 1 | 2 | 0 | 1 | 1 | 1 | NoOC: Illness *n* = 4, wrong phase *n* = 1  COC: Illness *n* = 3 |
| FM | 0 | 1 | 1 | 2 | 0 | 1 | 1 | 1 | NoOC: Illness *n* = 4, wrong phase *n* = 1  COC: Illness *n* = 3 |
| BM | 0 | 1 | 1 | 2 | 0 | 1 | 1 | 1 | NoOC: Illness *n* = 4, wrong phase *n* = 1  COC: Illness *n* = 3 |
| REE | 0 | 1 | 1 | 2 | 0 | 1 | 1 | 1 | NoOC: Illness *n* = 4, wrong phase *n* = 1  COC: Illness *n* = 3 |
| RER | 0 | 1 | 1 | 2 | 0 | 1 | 1 | 1 | NoOC: Illness *n* = 4, wrong phase *n* = 1  COC: Illness *n* = 3 |
| Absolute V̇O_2PEAK_ | 0 | 2 | 3 | 2 | 0 | 1 | 1 | 1 | NoOC: Illness *n* = 5, wrong phase *n* = 2  COC: Illness *n* = 3 |
| Relative V̇O_2PEAK_ | 0 | 2 | 3 | 2 | 0 | 1 | 1 | 1 | NoOC: Illness *n* = 5, wrong phase *n* = 2  COC: Illness *n* = 3 |
| E2 | 0 | 1 | 1 | 3 | 0 | 1 | 0 | 1 | NoOC: Illness *n* = 3, wrong phase, *n* = 1, failed blood draw *n* = 1  COC: Illness *n* = 2 |
| P4 | 0 | 1 | 1 | 3 | 0 | 1 | 0 | 1 | NoOC: Illness *n* = 3, wrong phase, *n* = 1, failed blood draw *n* = 1  COC: Illness *n* = 2 |
| fT3 | 0 | 1 | 1 | 3 | 0 | 1 | 0 | 1 | NoOC: Illness *n* = 3, wrong phase, *n* = 1, failed blood draw *n* = 1  COC: Illness *n* = 2 |
| UnAG | 0 | 1 | 1 | 3 | 0 | 1 | 0 | 1 | NoOC: Illness *n* = 3, wrong phase, *n* = 1, failed blood draw *n* = 1  COC: Illness *n* = 2 |
| AG | 0 | 1 | 1 | 3 | 0 | 1 | 0 | 1 | NoOC: Illness *n* = 3, wrong phase, *n* = 1, failed blood draw *n* = 1  COC: Illness *n* = 2 |
| EA | 1 | 2 | 2 | 2 | 1 | 1 | 1 | 1 | NoOC: Illness *n* = 4, wrong phase *n* = 1, missing diary *n* = 1, personal reasons *n* = 1  COC: Illness *n* = 2, missing diary *n* = 1, personal reasons *n* = 1 |

LUT_PRE_: luteal phase pre-measurements; LUT_POST_; luteal phase post-measurements; FOL_PRE_: follicular phase pre-measurements; FOL_POST_: follicular phase post-measurements; ACT_PRE_: active phase pre-measurements; ACT_POST_: active phase post-measurements; INACT_PRE_: inactive phase pre-measurements; INACT_POST_: inactive phase post-measurements; FFM; fat-free mass; FM: fat mass; BM: body mass; REE: resting energy expenditure; RER: respiratory exchange ratio; V̇O_2PEAK_: peak oxygen uptake; E2: estradiol; P4: progesterone; fT3: triiodothyronine; UnAG: unacylated ghrelin; AG: acylated ghrelin; EA: energy availability.

**Results of the sensitivity analysis**

**Supplemental Table 3.** Body composition, resting metabolism, aerobic performance, and hormones before and after the MIET intervention in the NoOC group and pooled sample after the sensitivity analysis excluding a participant with lengthened menstrual cycle, measurements completed at luteal or active phases.

|  | **NoOC** | | **Pooled sample** | | **P-value** | | | |
| --- | --- | --- | --- | --- | --- | --- | --- | --- |
| **A** | **LUT_PRE_** | **LUT_POST_** | **LUT/ACT_PRE_** | **LUT/ACT_POST_** | **Group** | **Time** | **Time × Group** |  |
| **Body composition** | *n* = 16 | *n* = 15 | *n* = 24 | *n* = 22 |  |  |  |  |
| FFM (kg) | 47.3 ± 3.9 | 47.3 ± 4.2 | 48.1 ± 4.8 | 48.0 ± 5.3 | 0.220 | 0.976 | 0.846 |  |
| FM (kg) | 19.5 ± 6.5 | 19.5 ± 6.5 | 19.4 ± 5.9 | 19.3 ± 5.8 | 0.943 | 0.380 | 0.749 |  |
| BM (kg) | 66.8 ± 8.5 | 66.8 ± 8.7 | 67.6 ± 8.0 | 67.3 ± 8.1 | 0.480 | 0.225 | 0.466 |  |
| **Resting metabolism** | *n* = 16 | *n* = 15 | *n* = 24 | *n* = 22 |  |  |  |  |
| Absolute REE (kcal·d^-1^) | 1463 ± 131 | 1430 ± 165 | 1454 ± 129 | 1440 ± 161 | 0.881 | 0.979 | 0.163 |  |
| RER (V̇O_2_/V̇CO_2_) | 0.85 ± 0.05 | 0.86 ± 0.05 | 0.85 ± 0.05 | 0.84 ± 0.04 | 0.296 | 0.250 | 0.147 |  |
| **Aerobic performance** | *n* = 16 | *n* = 14 | *n* = 24 | *n* = 21 |  |  |  |  |
| V̇O_2PEAK_  (mL∙kg^-1^∙min^-1^) | 37.3 ± 4.5 | 37.8 ± 4.0 | 37.7 ± 4.2 | 38.4 ± 4.5 | 0.600 | **0.006*** | 0.353 |  |
| V̇O_2PEAK_ (L∙min^-1^) | 2.50 ± 0.30 | 2.56 ± 0.31 | 2.57 ± 0.31 | 2.62 ± 0.34 | 0.195 | **0.039*** | 0.228 |  |
| **Female sex hormones** | *n* = 16 | *n* = 15 | *n* = 24 | *n* = 22 |  |  |  |  |
| E2 (pmol∙L^-1^) | 472.0 (408.0–704.8) | 485.0 (338.0–580.0) | 413.0 (187.8–628.0) | 387.5 (123.1–545.3) | **<0.001*** | 0.910 | 0.903 |  |
| P4 (nmol∙L^-1^) | 27.5 (20.9–35.6) | 26.2 (23.3–30.1) | 21.1 (1.1–33.3) | 23.5 (1.4–27.9) | **<0.001*** | 0.589 | 0.382 |  |
| **Metabolic hormones** | *n* = 16 | *n* = 15 | *n* = 24 | *n* = 22 |  |  |  |  |
| fT3 (pmol∙L^-1^) | 5.0 (4.6–5.2) | 4.7 (4.5–5.2) | 5.0 (4.7–5.5) | 4.9 (4.5–5.4) | **0.002*** | 0.760 | 0.483 |  |
| Leptin (ng·mL^−1^) | 15.4 (11.1–22.7) | 17.3 (9.6–29.1) | 15.2 (11.1–23.8) | 16.5 (10.6–29.5) | 0.751 | 0.859 | 0.583 |  |
| UnAG (pg·mL^−1^) | 315.2 (195.0–485.8) | 298.0 (195.4–407.0) | 364.1 (198.9–503.0) | 289.6 (185.2–428.7) | 0.644 | 0.057 | 0.104 |  |
| AG (pg·mL^−1^) | 43.4 (37.1–72.4) | 50.9 (36.2–123.1) | 43.4 (38.9–103.3) | 57.6 (37.9–146.2) | 0.568 | 0.994 | 0.599 |  |

LUT_PRE_: luteal phase pre-measurements; LUT_POST_; luteal phase post-measurements; ACT_PRE_: active phase pre-measurements; ACT_POST_: active phase post-measurements; FFM: fat-free mass; FM: fat mass; BM: body mass; REE: resting energy expenditure; RER: respiratory exchange ratio: V̇O_2PEAK_: peak oxygen uptake; E2: estradiol; P4: progesterone; fT3: free triiodothyronine; unAG: unacylated ghrelin; AG: acylated ghrelin ‘*’ indicates a main effect of time or group, *p* ≤ 0.05.

**Supplemental Table 4.** Results after sensitivity analysis for REE from A) follicular/inactive_PRE_ to follicular/inactive_POST,_ in the COC group and the pooled sample, excluding one participant based on boxplot analysis_,_ and for unacylated ghrelin from B) luteal/active_PRE_ to luteal/active_POST_ and C) follicular/inactive_PRE_ to follicular/inactive_POST_ in the COC group and the pooled sample, excluding one participant with Z-score of ±3.

|  | **COC** | | **Pooled sample** | |  |  |  |
| --- | --- | --- | --- | --- | --- | --- | --- |
| **A** | **INACT_PRE_** | **INACT_POST_** | **FOL/INACT_PRE_** | **FOL/INACT_POST_** | **Group** | **Time** | **Time × Group** |
|  | *n* = 7 | *n* = 6 | *n* = 23 | *n* = 21 |  |  |  |
| REE (kcal·d^-1^) | 1478 ± 196 | 1519 ± 157 | 1421 ± 181 | 1420 ± 168 | 0.156 | 0.639 | 0.318 |
| REE_ADJ_ |  | | | | 0.390 | 0.873 | 0.461 |
| **B** | **ACT_PRE_** | **ACT_POST_** | **LUT/ACT_PRE_** | **LUT/ACT_POST_** | **Group** | **Time** | **Time × Group** |
|  | *n* = 7 | *n* = 6 | *n* = 24 | *n* = 22 |  |  |  |
| UnAG (pg·mL^−1^) | 439.3 (261.9–505.6) | 304.1 (220.8–602.6) | 357.2 (198.9–494.0) | 289.6 (207.7–428.7) | 0.439 | 0.195 | 0.505 |
| **C** | **INACT_PRE_** | **INACT_POST_** | **FOL/INACT_PRE_** | **FOL/INACT_POST_** | **Group** | **Time** | **Time × Group** |
|  | *n* = 7 | *n* = 6 | *n* = 23 | *n* = 20 |  |  |  |
| UnAG (pg·mL^−1^) | 393.4 (257.9–498.9) | 279.2 (200.9–581.0) | 341.5 (295.0–472.8) | 247.1 (181.5–505.6) | 0.397 | 0.114 | 0.577 |

INACT_PRE_: inactive phase pre-measurements; INACT_POST_: active phase post-measurements; FOL_PRE_: follicular phase pre-measurements; FOL_POST_: follicular phase post-measurements; REE: resting energy expenditure; REE_ADJ_: resting energy expenditure adjusted for FFM and FM; ACT_PRE_: active phase pre-measurements; ACT_POST_: active phase post-measurements; LUT_PRE_: luteal phase pre-measurements; LUT_POST_; luteal phase post-measurements; unAG: unacylated ghrelin.
